# Bio-priming of banana tissue culture plantlets with endophytic *Bacillus velezensis* EB1 to improve Fusarium wilt resistance

**DOI:** 10.3389/fmicb.2023.1146331

**Published:** 2023-03-16

**Authors:** Dandan Xiang, Xiaofang Yang, Bojing Liu, Yuanqi Chu, Siwen Liu, Chunyu Li

**Affiliations:** ^1^Key Laboratory of South Subtropical Fruit Biology and Genetic Resource Utilization, Ministry of Agriculture and Rural Affairs, Guangdong Provincial Key Laboratory of Tropical and Subtropical Fruit Tree Research, Institute of Fruit Tree Research, Guangdong Academy of Agricultural Sciences, Guangzhou, China; ^2^College of Horticulture, Sichuan Agricultural University, Chengdu, China

**Keywords:** endophyte, *Bacillus*, Fusarium wilt of banana, biological control, banana tissue culture plantlets

## Abstract

Tissue culture techniques have been routinely used for banana propagation and offered rapid production of planting materials with favorable genotypes and free of pathogenic microorganisms in the banana industry. Meanwhile, extensive scientific work suggests that micropropagated plantlets are more susceptible to *Fusarium oxysporum* f. sp. *cubense* (*Foc*), the deadly strain that causes Fusarium wilt of bananas than conventional planting material due to the loss of indigenous endophytes. In this study, an endophytic bacterium *Bacillus velezensis* EB1 was isolated and characterized. EB1 shows remarkable *in vitro* antagonistic activity against *Foc* with an inhibition rate of 75.43% and induces significant morphological and ultrastructural changes and alterations in the hyphae of *Foc*. Colony-forming unit (c.f.u.) counting and scanning electron microscopy (SEM) revealed that EB1 could colonize both the surface and inner tissues of banana tissue culture plantlets. Banana tissue culture plantlets of late rooting stage bioprimed with EB1 could efficiently ward off the invasive of *Foc*. The bio-priming effect could maintain in the acclimatized banana plants and significantly decrease the disease severity of Fusarium wilt and induce strong disease resistance by manipulating plant defense signaling pathways in a pot experiment. Our results provide the adaptability and potential of native endophyte EB1 in protecting plants from pathogens and infer that banana tissue culture plantlets bio-priming with endophytic microbiota could be a promising biological solution in the fight against the Fusarium wilt of banana.

## Introduction

As the most important fruit in the world and the major staple crop in more than 130 countries across the tropical belt, banana (*Musa* spp.) production contributes significantly to income and food security (Kema et al., 2021). However, the banana industry is under severe threat from Fusarium wilt, the most destructive disease of banana in history whose causal agent is *Fusarium oxysporum* f. sp. *cubense* (*Foc*). *Foc* is composed of different evolutionary lineages and at least 24 vegetative compatibility groups (VCGs). *Foc* race 1 wiped out the highly susceptible Gros Michel (*Musa* AAA) variety in Central America in the mid-twentieth century (O'Donnell et al., 2009; Staver et al., 2020). The plague caused by *Foc* race 1 was mitigated by gradually adopting a resistant cultivar Cavendish (*Musa* AAA) as a replacement for Gros Michel (Dita et al., 2018). The recent emergence of *Foc* tropical race 4 (*Foc* TR4), the most destructive and uncontrollable pathogen of banana, to which Cavendish and many other cultivars are highly susceptible, has created havoc on banana production worldwide again (Dita et al., 2018). Ever since it was first reported to destroy the Cavendish-based banana industry in the 1960s in Taiwan, *Foc* TR4 has expanded across Southeast Asia, the Middle East, Africa, and most recently has been reported in Colombia and is present in 27 countries where thousands of hectares have been affected in the past several years (Ordonez et al., 2015; Galvis, 2019). A recent projection by the Food and Agriculture Organization of the United Nations (FAO) estimated that the inexorable spread of *Foc* TR4 would lead to a 2.0% drop in global output, 240,000 direct jobs loss, and induce a 9.2% rise in the global reference price for bananas by 2028 (Altendorf, 2019).

The management of Fusarium wilt is particularly challenging due to several conspiring factors. First, as a soil-borne fungus, *Foc* can survive in the soil in the form of chlamydospore for up to 30 years even in the absence of host plants and be dispersed through diverse ways (i.e., infecting plant material, soil, water, and others) (Cook et al., 2015; Dita et al., 2018). Second, the only effective measure to manage this disease is stated frequently as planting resistant cultivars, but resistant cultivars might not meet the current market demand and may be overcome by continually emerging pathogens (Ploetz, 2015). The latter cultivated banana is almost exclusively of the *Foc* TR4 susceptible Cavendish for its export properties, which facilitates the dispersion of the disease worldwide. Third, as a typical vascular wilt disease, *Foc* can escape from contacting with non-contact fungicides, non-endophytic biological control agents (BCAs), and other control measures once it penetrates the host plant (Bubici et al., 2019). Thus, it is almost impossible to eliminate the disease incidence once the field gets contaminated with *Foc*. Therefore, highly efficient and sustainable strategies should be implemented to alleviate the influences of Fusarium wilt on susceptible varieties and to improve the durability of available resistant varieties.

In recent years, the use of BCAs for the control of many plant diseases including the Fusarium wilt of banana has gained great interest as an alternative to chemical application. Among the BCAs, Among the BCAs, the pivotal role of endophyte in the health and fitness of their host plants has become evident only in recent years (Papik et al., 2020; Matsumoto et al., 2021). Endophytes refer to microbes that colonize internally in different plant tissues and perform mutualistic symbiotic associations with their hosts (Papik et al., 2020). Their unique ecological niches similar to that of vascular wilt pathogens make them better targets for biocontrol agents against wilt disease than their rhizospheric counterparts (Strobel and Daisy, 2003; Eljounaidi et al., 2016). As the second microbiological layer of plant defense, endophytes can defend plants from biotic stresses either by showing direct antagonistic activity such as parasitism, antibiosis, and competition or by inducing indirect antagonism effects (induced systemic resistance, ISR) in host plants to an array of phytopathogens (Dini-Andreote, 2020; Dubey et al., 2020). Several studies have shown that endophytic microbes may serve as environmentally safe measures to combat Fusarium wilt of banana (Cao et al., 2005; Bubici et al., 2019; Gómez-Lama Cabanás et al., 2021; Savani et al., 2021; Zhang et al., 2022). Applications of endophytic *Trichoderma asperellum Prr2* (Thangavelu and Gopi, 2015), *Pseudomonas aeruginosa* (Yu et al., 2010), *Pseudomonas* sp. UPMP3, and *Burkholderia* sp. UPMB3 (Mohd Fishal et al., 2010) have reduced the disease incidence of Fusarium wilt in banana significantly under greenhouse and field conditions.

Nowadays, the most common application strategies of endophytes in agricultural systems are adding them directly into the soil and preparing them as seed-coating agents, which are rather inefficient in practice. Thus, it is imperative to explore alternative strategies for endophyte application (Dubey et al., 2020). Even more significant is the fact that, unlike most other seed plants, the propagation of banana is mainly dependent on tissue culture with all microorganisms eliminated during the micropropagation process under strict aseptic conditions. The regenerated plants are, therefore, particularly vulnerable when transferred directly to natural conditions with multiple environmental stresses (Lian et al., 2009; Soumare et al., 2021). In this sense, the establishment of beneficial interactions between explants and beneficial microbes to offer protection for young host plantlets against environmental stress in field conditions might represent a valuable approach to efficiently solve those restrictions (Soumare et al., 2021). Unfortunately, only few studies have reported inoculation with endophytes in banana tissue culture plantlets during the rooting or acclimatization stages (Guez-Romero et al., 2008; Lian et al., 2009; Kavino and Manoranjitham, 2018). As the key components for achieving sustainable agriculture, the interactions between plants, fungi, and endophytes in tissue culture plantlets have not been sufficiently studied. In the present study, a bacterial endophytic strain EB1 was isolated from a healthy banana plant in a wilt-diseased banana field in Dongguan, Guangdong Province, China (23.045315° N, 113.546177° E). We critically aimed to decipher (i) the phylogenetic, genomic, and antagonistic effect of EB1 against *Foc* by *in vitro* test and (ii) how EB1 modulates the resistance of banana plants against *Foc* by using a banana plant–EB1system created by inoculating banana tissue culture plantlets with EB1 at the end of rooting stage. Our study was designed to lend new insights into the sophisticated mechanisms of host plants–endophytes interactions for coping with environmental stresses and to provide potential strategies to control the Fusarium wilt of banana.

## Materials and methods

### Strain, media, and cultural conditions

Wild-type *Foc* TR4 strain II5 (VCG01213) was cultivated on a potato dextrose agar (PDA) plate at 28°C and used in this study. Isolated endophytic bacteria were inoculated in Luria-Bertani (LB) agar (Sangong Co., Ltd., Shanghai, China) plates. Basal Murashige and Skoog (MS) medium was used for tissue culture experiments.

### Isolation and selection of endophytic bacteria against *Foc* TR4 from healthy banana plant

The healthy banana plants used in this study were collected from a wilt-diseased banana field in Dongguan, Guangdong Province, China (23.045315° N, 113.546177° E). Banana plant samples were washed with tap water thoroughly to remove the airborne counterparts and soaked in 75% (v/v) ethanol for 1 min, 0.1% (v/v) NaClO for 15 min, followed by being rinsed 5 times with sterile water to deplete epiphytic microorganisms in aseptic conditions. Ten grams of plant tissue was weighed and ground with 20 ml sterilized distilled water premixed with sterilized quartz sand using a sterilized mortar and pestle for 5 min. Aliquots of 1 ml of the resulting suspension were diluted using a serial dilution method and spread evenly on an LB agar plate and incubated at 28°C for 5 days. All culturable bacterial colonies were purified and selected based on their morphological characteristics. The antagonistic efficacies of these endophytic bacterial isolates were evaluated against *Foc* TR4 by a dual-culture experiment (Fan et al., 2019). One actively growing agar plug (5 mm diameter) of *Foc* TR4 was placed on the center of a fresh PDA plate. Then, 10 μL-drop of each isolate from an overnight culture (OD_600_ = 1.0) was uniformly inoculated 2.0 cm away from the fungal inoculum. Plates inoculated only with *Foc* TR4 plug were served as control. Plates were incubated at 28°C for 5 days and recorded with a Canon EOS 77D camera (Canon, Tokyo, Japan) with the same parameters, and the surface area of the mycelia was measured using the Image J software (Image J, NIH, USA). The inhibitory effect was evaluated by calculating the percentage of area inhibition using the following formula: (Sc - St)/Sc × 100, where Sc and St represent the growth area of *Foc* TR4 in the control and treated plates, respectively. The experiment was repeated 3 times, with 4 replicates each time. Strain EB1 was isolated through the above screening and stored at −80°C with glycerol (50%, v/v). The antifungal efficiency of EB1 against *Foc* TR4 was further measured by observing the morphology and ultrastructure characteristics of *Foc* TR4 in the dual-culture experiment by applying a scanning electron microscope (SEM, Hitachi Model S-3400N, Hitachi, Tokyo, Japan) and a transmission electron microscope (TEM, Hitachi HT7700, Hitachi, Tokyo, Japan).

### Whole-genome sequencing of EB1

Overnight bacterial cultures of EB1 in LB broth were collected, centrifuged at 3,000 rpm for 15 min, and washed two times with sterile PBS buffer (50 μM, pH = 7.4). Whole-Genome Sequencing of EB1 was performed using a combination of the Oxford Nanopore Technologies (ONT) GridION platform (Oxford Nanopore Technologies Ltd, Oxford, UK) and Illumina MiSeq platform (Illumina MiSeq PE300, Illumina, USA) by Gene Denovo Biotechnology Co. (Guangzhou, China). DNA was extracted from Qiagen's DNeasy UltraClean Microbial Kit (Qiagen GmbH, Hilden, Germany) and its quality and concentrations were determined using a Nanodrop spectrophotometer (NanoDrop, Wilmington, DE, USA) and Qubit Fluorometer (Thermo Fisher Scientific, MA, USA). For ONT sequencing, library preparation was conducted according to the manufacturer's protocol of the SQK-LSK109 sequencing kit (Oxford Nanopore Technologies Ltd., Oxford, UK). For Illumina sequencing, genomic DNA (gDNA) was fragmented and a paired-end library with an average DNA insert size of 300–400 bp was constructed using Illumina TruSeq Nano DNA Library Prep Kit (Illumina). The assembled sequences were deposited in the NCBI (BioProject ID: PRJNA807456). The components of coding genes, noncoding RNA (ncRNA), and functional annotation were analyzed using a range of databases including the non-redundant protein database (Nr), SwissProt, Cluster of Orthologous Groups (COGs), and Kyoto Encyclopedia of Genes and Genomes (KEGG). Gene clusters for the biosynthesis of secondary metabolites were identified by using antiSMASH.

### Phylogenetic analysis of EB1

The 16S rDNA sequence of strain EB1 derived from the EB1 genome was aligned with an NCBI 16S ribosomal RNA sequences database by Nucleotide BLAST (https://blast.ncbi.nlm.nih.gov/Blast.cgi), and 16S rRNA gene sequences closest to the isolates (98% sequence homology) were recovered for further phylogenetic analysis. Strain EB1 was subjected to phylogenetic analysis using MEGA version 7 (University, Pennsylvania, PA, USA) based on a full-length 16S ribosomal RNA (16S rRNA) sequence, and a phylogenetic tree was constructed using the neighbor-joining method. The reliability of this resulting tree was evaluated by the bootstrap method with 1,000 replications.

### Colonization capacity of EB1 on banana tissue culture plantlets

Uniformly grown banana tissue culture plantlets [“Cavendish” banana (AAA) cv. “Brazilian”] of rooting stage were surface sterilized in 75% (v/v) ethanol for 1 min and 0.1% (v/v) NaClO for 15 min, rinsed with sterile water for 5 times, air-dried, and then transferred and grown vertically in tissue culture flasks containing 100 ml of cooled-down MS. Four plantlets were transferred to each flask. For EB1 inoculation, overnight culture of EB1 in LB broth was harvested, centrifuged, and washed in liquid MS twice, and resuspended in MS to a final optical density (OD_600_) = 0.2. Each flask was inoculated with 0.1 ml of the bacterial suspension (~10^6^ colony-forming unit, c.f.u.) or 0.1 ml MS by pipetting to the rhizosphere of banana tissue culture plantlets and cultured at 22°C and 16/8 h light/dark cycle. The colonization and reproduction of EB1 on the plantlets were quantified each day over a period of 7 days. Total c.f.u. values of EB1 were quantified per the programs described previously (de Zélicourt et al., 2018; Berlanga-Clavero et al., 2022). Briefly, 1.0 g root and pseudostem tissues of the banana tissue culture plantlets were sampled and gently washed by dipping in the distilled water to remove non-attached bacteria cells at the same time each day (±2 h). Each sample was transferred to a 2-ml microcentrifuge tube with 1 ml PBS buffer, sonicated on ice for 1 min, and vortexed for 10 min, and 100 μl of the resulting suspensions were spread on LB agar plates after a 10-time dilution. The c.f.u. was counted after overnight incubation at 28°C, and the total c.f.u. was normalized per gram of root or pseudostem. To explore the interactions between strain EB1 and *Foc in planta*, an additional experiment was conducted in banana tissue culture plantlets by pipetting 0.1 ml *Foc* spore suspension (1 × 10^8^ spores/L) or 0.1 ml MS to the rhizosphere of banana tissue culture plantlets after prior inoculation with EB1 for 3 days and cultured at 22°C and 16:8 h light/dark cycle for another 4 days. The experiment was conducted in triplicate, with at least eight plants per treatment. After 7 days of successive culture, SEM was used to observe the distributions of EB1 and the interactions between EB1 and *Foc* on the banana tissue plantlets.

### Biocontrol efficacy of EB1 on banana plantlets

The above banana tissue culture plantlets and symbionts (banana tissue culture plantlets colonized with EB1) after 7 days of successive culture were subjected to hardening for 10 days by transferring into pots with sterilized planting soil (40 × 19 × 15 cm pots, ca. 2.0 kg soil each). Then the biocontrol efficacy of EB1 on banana plantlets was investigated in greenhouse experiments with the acclimatized banana plants (Supplementary Figure S1). Three treatments including EB1 only, TR4 only, EB1+ TR4, and control were applied in the pot. The banana plants were inoculated with or without *Foc* TR4 isolates at the concentration of 1,000 conidia/g soil, with a temperature ranging from 25 to 35°C. Three plantlets were grown in each pot and at least 20 plantlets were included in each treatment. In addition, due to the lethality of plantlets in the TR4 only, EB1 + TR4 groups, 60 plantlets were employed in each group to ensure sufficient plant material. Plant survival rates were recorded every 10 days, and observations on morphological characters such as plant height (cm) and fresh weight of shoot and root (g) were conducted after 60 days of planting. The disease index of each plantlet was assessed according to the rating scale of 0–4: 0 = no symptom; 1 = some brown spots in the inner rhizome; 2 = < 25% of the inner rhizome show browning; 3 = up to 75% of the inner rhizome show browning; and 4 = entire inner rhizome and pseudostem show dark brown, dead (Liu S. et al., 2020). Harvested banana plant tissues were stored at −80°C pending for further analysis of defense-related enzymes and genes.

### RNA extraction and gene expression analysis by RT-qPCR

Total RNA was extracted from the frozen banana plant using SteadyPure Plant RNA Extraction Kit (Accurate Biotechnology Co., Ltd., Hunan, China**)** following the manufacturer's instructions. HiScript II One Step qRT-PCR SYBR Green kit (Vazyme Biotech, Nanjing, China) was employed for qRT-PCR assays according to the manufacturer's instructions. First-strand cDNA was prepared by reverse transcription from 1 μg of DNA-free total RNA in a final reaction volume of 20 μl. RT-qPCR was conducted using a QuantStudio 5 Real-Time PCR System (Applied Biosystems, CA, USA) in four replicates. The *qTUB* gene (banana) was used as a reference for data normalization, and the target genes were amplified using the primer sets listed in Supplementary Table S1. The relative transcript abundance of each gene was estimated using the 2^−ΔΔ*Ct*^ method (Livak and Schmittgen, 2001).

### Statistical analyses

All statistical analyses were performed using the SPSS 20.0 statistical software package (SPSS, Chicago, IL, USA). Data were analyzed using Student's *t*-test and one-way ANOVA test after being verified for normality and homogeneity of variance with Kolmogorov-Smirnov and Levene's tests. Cases with *p*-values of < 0.05 were considered statistically significant.

## Results

### EB1 shows strong inhibitory efficiency against *Foc* TR4

**Figure 1:**
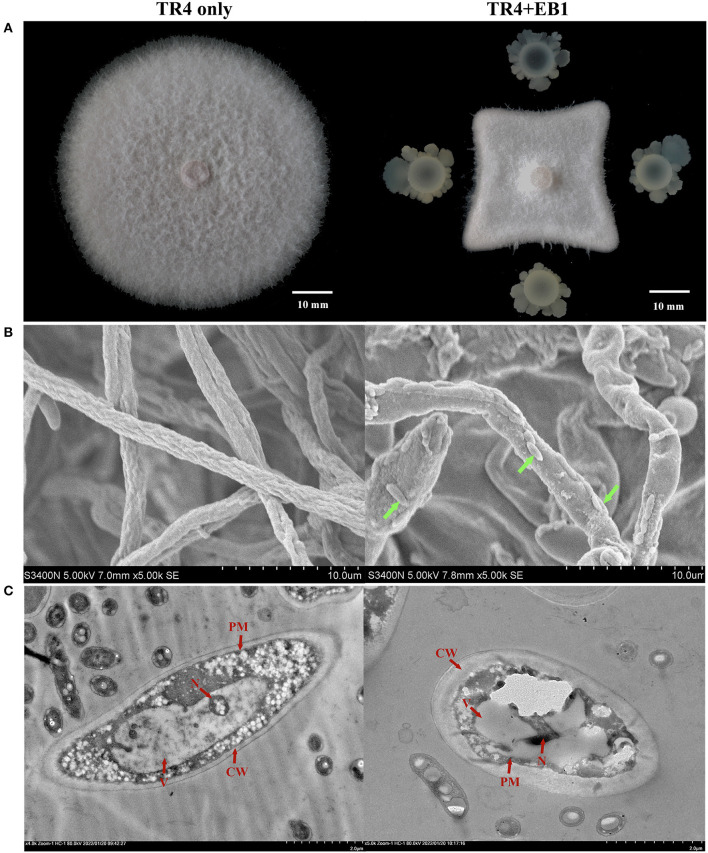
The antagonistic potential of strain EB1 against *Fusarium oxysporum* f. sp. *cubense* tropical race 4 (*Foc* TR4). **(A)** The antagonistic potential of EB1 against TR4 *in vitro* with a dual-culture experiment. Morphological (marked by green arrow heads) and ultrastructural changes (marked by red arrow heads) of TR4 after the confrontation with EB1 were scrutinized by scanning electron microscopy (SEM) **(B)** and transmission electron microscope (TEM) **(C)**, respectively.

The morphological observation was preliminarily carried out for strain EB1. It was found that the colony of EB1 on LB medium was dry and round with irregular protrusions at the margin, showing the typical characteristics of *Bacillus* species (Supplementary Figure S2A). The cells were short rod-shaped structures and ~1.2–1.6 μm in length, 0.6–0.7 μm in width, as revealed by SEM (Supplementary Figure S2B). EB1 showed strong inhibitory activities with the inhibition rates of mycelium growth area 75.43% against *Foc* TR4 (Figure 1A) and other Fusarium pathogens (Supplementary Figure S3) during co-cultivation compared to control. To confirm the antagonistic activity of EB1, the morphological and ultrastructural changes of *Foc* TR4 after a confrontation with EB1 were scrutinized by SEM (Figure 1B) and TEM (Figure 1C). The untreated hyphae of *Foc* TR4 appeared straight, uniform, and well-developed tube-like structure in shape under SEM. Conversely, phenotypes of abnormalities were noted in fungal hyphae co-culture with EB1. Severe forms of abnormalities, including highly deformed, irregular distorted, inflated were observed in fungal hyphae. TEM micrographs of untreated fungal hyphae had a well-defined cell wall (CW), intact plasma membrane (PM), and normal cytoplasm containing an intact nucleus and all organelles. In reverse, noticeably disruptions such as loss of cellular integrity, thickened CW, evident plasmolysis, serious vacuolation, invaginated PM, abnormal architecture of the nucleus and degenerated organelles were observed in EB1 treated hyphae. The results indicated that the morphology and structural integrity of the treated fungal were dramatically affected during co-culture with EB1.

### Genome sequence assembly and general features of EB1

**Figure 2:**
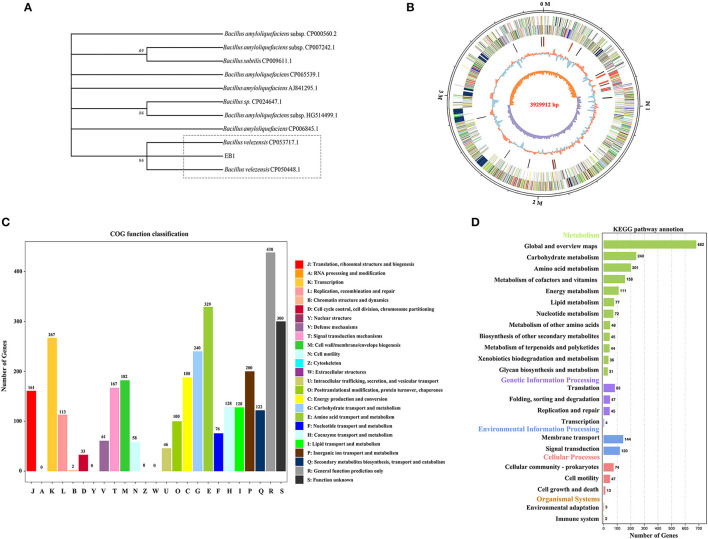
Phylogenetic and genomic analyses of strain EB1. **(A)** Phylogenetic trees of strain EB1 based on 16S rRNA gene. The tree was constructed using the MEGA software. The level of bootstrap support (1,000 repetitions) was indicated at all nodes. **(B)** Graphical circular map of EB1 genome. The distribution of the circle from the outermost to the center is (i) scale marks of the genome; (ii) protein-coding genes on the forward strand; (iii) protein-coding genes on the reverse strand; (iv) tRNA (black) and rRNA (red); (v) GC content; (vi) GC skew. **(C)** The COG annotation of strain EB1 genome. **(D)** The KEGG pathway annotation of strainEB1 genome.

The whole genome of EB1 was sequenced and analyzed, through which the 16S rRNA region was extracted, and which was 1,404 bp in length. EB1 was identified as *Bacillus velezensis* based on a phylogenetic tree constructed from the 16S rRNA gene (Figure 2A). The complete genome sequence of EB1 was deposited in GenBank under accession number CP093218. Accordingly, the genome of EB1 consists of a single circular chromosome of 3,929,912 bp, with an average of 46.5% GC content and a clear GC skew transition (Figure 2B). All predicted 3,622 open reading frames (ORFs) with a maximum *E*-value of 1.0 E-5 were subjected to annotation analysis by comparing with Nr, SwissProt, COG, and KEGG databases, and a total of 3,606 candidate genes had annotation information. The overall functional annotation is depicted in Supplementary Figure S4. A total of 2,756 genes were categorized into 21 functional groups using COG analysis (Figure 2C). Three main functional gene classes were revealed in the results: amino acid transport and metabolism (329 genes), transcription (267 genes), and carbohydrate transport and metabolism (240 genes), representing 30.33% of the predicted genes in the COG analysis. Other clusters of represented genes involved in inorganic ions transport and metabolism (200 genes), energy production and conversion (188 genes), cell wall/membrane/envelope biogenesis (182 genes), signal transduction (167 genes), and translation, ribosomal structure and biogenesis (161 genes) account for 32.58% of predicted genes. In addition, a high proportion of predicted genes (26.78%) involved in general function prediction only and function unknown is poorly characterized. A total of 2,250 genes were mapped to 5 KEGG branches, including metabolism, genetic information processing, environmental information processing, cellular processes, and organismal systems, and among which, a high proportion of the annotated genes were assigned to metabolism, especially the pathways belonging to global and overview maps (682 genes), carbohydrate metabolism (240 genes), and amino acid metabolism (201 genes) (Figure 2D). Twelve biosynthetic gene clusters (BGCs) involved in the biosynthesis of secondary metabolites including non-ribosomal peptides synthase (NRPS), bacteriocin-NRPS, trans-AT polyketide synthase (transatpks), type III polyketides synthase (t3pks), terpene, transatpks-nrps, lantipeptide, and other types of polyketide synthases (OtherKS) were identified in EB1 using AntiSMASH.

### EB1 showed strong colonization ability on the banana plantlets

**Figure 3:**
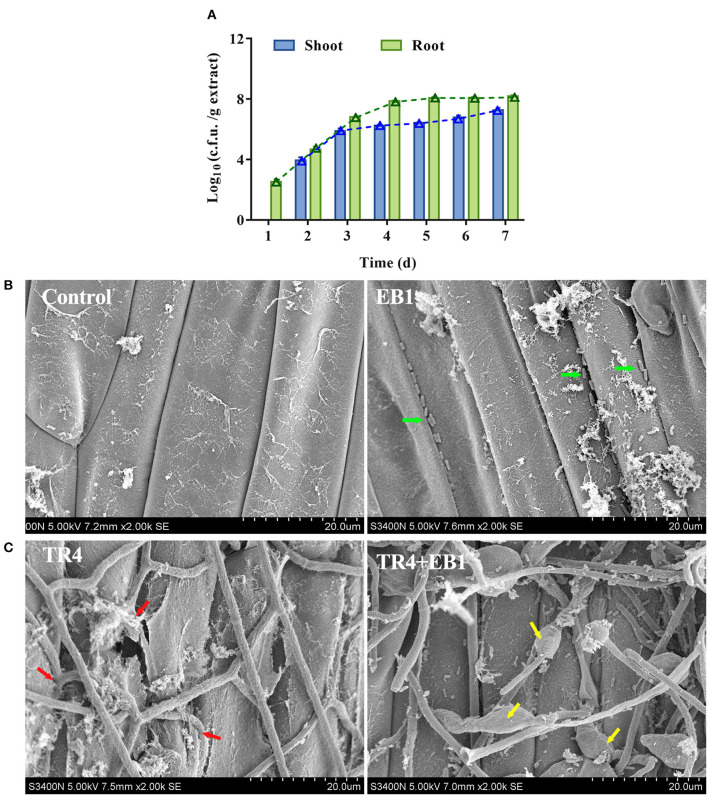
Strain EB1 showed strong colonization ability on the banana plantlets and provided protection against the infection of TR4. **(A)** EB1 dynamics (c.f.u. counts) in shoot and root extracts during the first 7 days after banana tissue culture plantlets bacterized. Bars represent average values ± SD of total c.f.u. in shoot and root extracts. Green and blue lines represent c.f.u. corresponding to the number of spores in shoot and root extracts, respectively. **(B)** Root colonization of strain EB1 visualized by scanning electron microscopy (SEM). Strain EB1 colonies occur in grooves between epidermal cells and intercellular space in root cells (marked by green arrow heads). **(C)** Strain EB1 inhibits the infection of TR4 *in planta*. TR4 seriously destroyed the surface structures of the root of banana tissue culture plantlets (marked by red arrow heads) and inoculation of EB1 prior to *Foc* treatment led to serious morphological deformities of *Foc* and the damaging effects by pathogen infection were alleviated (marked by yellow arrow heads).

The growth dynamics of the EB1 population during the 7 days after banana tissue plantlets treatment in tissue culture flasks were investigated in the root and shoot by c.f.u. counts and SEM (Figure 3). In the root, the colonization of EB1 numbered at 357 c.f.u./g on day 1 and increased to 1.20 × 10^8^ c.f.u./g following an algorithm by day 5 and remained at 1.53 × 10^8^ c.f.u./g by day 7. In contrast, in the case of shoot, no EB1 was detected on day 1, after which the colonization level slowly started to increase from 9.73 × 10^3^ c.f.u./g on day 2 to 1.96 × 10^7^ c.f.u./g by day 7 (Figure 3A). Using SEM, no colony could be observed in the root or shoot and the pant epidermal cells were integral and smooth in the control group (Figure 3B). In the EB1-inoculated group, small rod-shaped EB1 can be seen in grooves between epidermal cells and intercellular space in root cells (Figure 3B). To explore whether EB1 inhibits *Foc in planta*, an additional experiment was conducted in banana tissue culture plantlets by inoculating with *Foc* after prior inoculation with EB1 for 7 days. The SEM observation revealed that infection of *Foc* seriously destroyed the surface structures of the root of banana tissue culture plantlets (Figure 3C). However, inoculation of EB1 prior to *Foc* treatment led to serious morphological deformities of *Foc* and the damaging effects of pathogen infection were alleviated (Figure 3C).

### EB1 significantly promoted plant growth and conferred protection against *Foc* TR4

**Figure 4:**
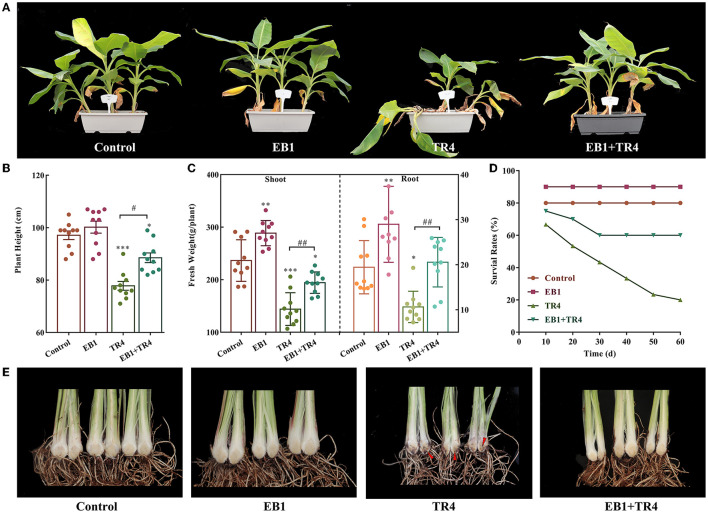
Strain EB1 enhances Fusarium wilt resistance and promoted banana growth in the pot experiment. Control, no inoculation; EB1, inoculation with EB1 only, TR4, inoculation with TR4 only; EB1 + TR4, inoculation of EB1 prior to *Foc* treatment. **(A)** Phenotypes of acclimatized banana plants after being primed with EB1 at the rooting stage of tissue culture plantlets. **(B–E)** Plant height **(B)**, biomass of shoots and roots **(C)**, survival rates **(D)**, and severity of Fusarium wilt of acclimatized banana plants under different treatments (marked by red arrow heads) **(E)**. All data are expressed as the mean ± SD of at least 10 replicate samples. *p < 0.05, **p < 0.01, and ***p < 0.001 indicate significant differences between the treatment groups and control group. ^#^*p* < 0.05 and ^##^*p* < 0.01 indicate significant differences between treatment groups.

As EB1 was pre-inoculated on the culture plantlets at the rooting stage, to evaluate whether the acclimatized banana plants have been primed by EB1, the plant growth, survival, and disease severity were inspected in banana plants. Overall, pre-inoculation with EB1 at the rooting stage significantly promoted plant growth and conferred protection against *Foc* TR4 compared with the non-inoculation groups (Figure 4). No differences were detected in the height of banana plants between the control and EB1 groups. By contrast, the heights of banana plants were significantly reduced when subjected to *Foc* TR4 infections (*p* < 0.001), and this inhibitory effect was dramatically reversed upon co-inoculation with EB1 (TR4 + EB1) (Figures 4A, B). Compared with control, EB1 inoculation (EB1) significantly increased the plant biomass in both above-ground (shoot) and below-ground (roots) by 1.22- (*p* = 0.002) and 1.49-fold (*p* = 0.007), respectively. Plants in the EB1 + TR4 treatment group also had increased biomass compared with those treated by *Foc* TR4 only (shoot: 1.35-fold, *p* = 0.002 and root: 1.94-fold, *p* = 0.004). Moreover, EB1 did not cause any mortality or disease symptoms in banana plants when inoculated alone and greatly enhanced the survival rates and reduced the disease severity caused by *Foc* TR4 in banana plants who had been pre-inoculated with EB1 prior to inoculation with *Foc* TR4 (EB1 + TR4) compared with those inoculated only with *Foc* TR4 (Figures 4D, E; Supplementary Figure S5). Overall, these findings reveal that EB1 is a bacterial endophyte of banana plants that efficiently suppresses Fusarium wilt caused by *Foc* TR4.

### EB1 manipulated the SA and JA pathways in banana plants

**Figure 5:**
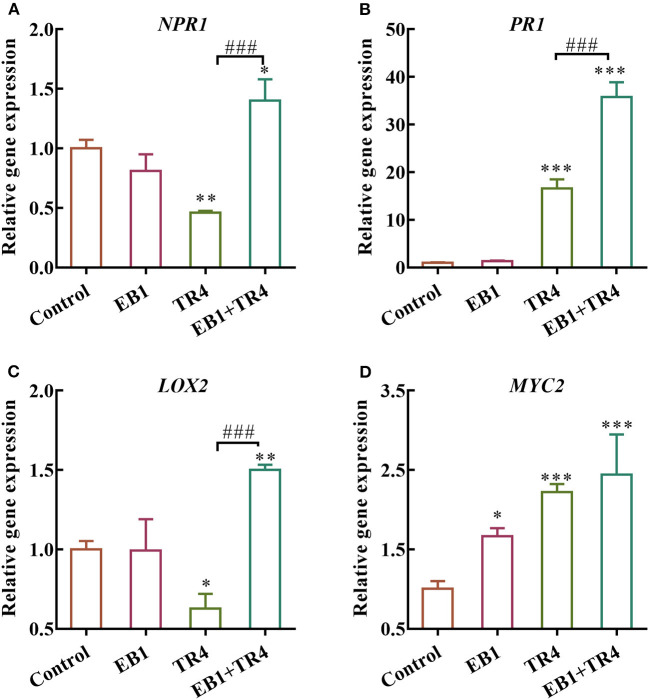
Effects of strain EB1 and TR4 inoculations on the expression of defense genes. The expression patterns of **(A)** *NPR1*, **(B)** *PR1*, **(C)** *LOX2*, and **(D)** *MYC2* were analyzed by RT-qPCR. All data are expressed as the mean ± SD (*n* = 4). *p < 0.05, **p < 0.01, and ***p < 0.001 indicate significant differences between treatment groups and control group. ^*###*^*p* < 0.001 indicate significant differences between treatment groups.

To further examine whether the EB1 could activate defense signaling in the banana plant, the expression patterns of the defense-related marker genes involved in SA and JA pathways including *NPR1, PR1, LOX2*, and *MYC2* were analyzed (Figure 5). Compared with the control, EB1 inoculation showed no induction in the expression of *NPR1* and *PR1* genes of the SA signaling pathway but showed an increase in the expression of the *MYC2* gene of the JA signaling pathway by 1.67-fold (*p* = 0.016). Whereas, the degree of expression changes in the *Foc* TR4 treatment group varied from gene to gene. *Foc* TR4 downregulated the expressions of *NPR1* and *LOX2* by 0.46- (*p* = 0.0024) and 0.63-fold (*p* = 0.016) and upregulated the expressions of *PR1* and *MYC2* by 16.62- (*p* < 0.001) and 2.23-fold (*p* < 0.001), respectively. Notably, both SA and JA signaling pathways were significantly upregulated by 1.40- (*p* = 0.015), 35.80- (*p* < 0.001), 1.50- (*p* = 0.0028), and 2.44-fold (*p* < 0.001) for *NPR1, PR1, LOX2*, and *MYC2*, respectively. The above results have indicated that EB1 primes the plants for enhanced immunity following a subsequent attack by *Foc* TR4.

## Discussion

Besides resistant cultivar breeding, BCA comprising endophytic bacteria has been considered another promising control strategy against Fusarium wilt that reached the field-testing stage (Bubici et al., 2019). However, their control efficacy has been always unstable due to the varying environmental conditions, accentuating the need to develop a new efficient strategy for endophytes to harness their maximum benefits (Dubey et al., 2020; Papik et al., 2020; Savani et al., 2021; Jana et al., 2022). Plant tissue culture is the main strategy for banana propagation with the advantages of high multiplication rates and the production of disease-free planting materials with high genetic fidelity and high standards of hygiene (Pegg et al., 2019). More recently, tissue-cultured banana plantlets are questioned to be more susceptible to Fusarium wilt than vegetative planting materials because the first three stages (Stage I: establishment of explants, Stage II: elongation and multiplication, and Stage III: rooting) of the plant tissue culture process are taken place in aseptic conditions without the possibility of interaction with beneficial microbes (Orlikowska et al., 2017). In this context, co-cultivation of the plant tissue culture plantlets with beneficial microorganisms may be indispensable to improve the adaptive ability of plantlets in the acclimation stage, which has accentuated the urgent to develop new technologies based on endophytes as microbial inoculants in tissue culture banana plantlets. Therefore, in the current study, the endophytic bacterial strain *B. velezensis* EB1 was isolated and selected for in-depth analysis based on its antifungal activity against *Foc* TR4 and strong colonization ability in banana plants to support a new approach using EB1 to control Fusarium wilt of banana by introducing into banana tissue culture plantlets at the end of rooting stage.

As one of the largest bacterial genera, *Bacillus* strains coexist with plants and are among the most studied microorganisms in the biological control of various plant diseases (Shafi et al., 2017; Fira et al., 2018). For instance, endophytic bacteria *B. mojavensis* and *B. cereus* exhibited potent inhibition activities against various rice Fusarium pathogens such as *F. proliferum, F. verticillioides*, and *F. fujikuroi* (Etesami and Alikhani, 2017). Consistent with these findings, the *in vitro* dual-culture experiment demonstrated that the growth of *Foc* TR4 could be significantly inhibited by EB1. Interestingly, considerable ultra-structural alterations such as wizened, flattened, thickened CW and plasmolysis that were observed in *Foc* TR4 cells at confronting with EB1 indicated that EB1 might be capable of producing antagonistic metabolites, penetrating into *Foc* TR4 cell, and leading to leakage of cytoplasm and disruption of internal organelles of *Foc* TR4. Accordingly, genes related to the biosynthesis of antifungal compounds such as lipopeptides and ketones, which were proven to inhibit hyphal extension and spore formation of phytopathogens, were identified in EB1 genome by the antiSMASH tool (Arrebola et al., 2010; Li et al., 2015).

Plants can act as a filter of microbial communities and select the right endophytes to maintain their normal growth and development (Dubey et al., 2020; Liu H. et al., 2020). Therefore, stable root colonization and persistence of BCAs in the plant is a key factor for their application in the biological management of microbial diseases (Shafi et al., 2017). Detections of EB1 inside both roots and shoots of banana tissue culture plantlets in our study supporting EB1 is capable of entering through the root system and migrating upwards into the pseudostem. EB1 is an endophyte isolated from banana pseudostem; thus, it is conceivable that it has evolved strategies for efficient adaptation to this niche. To determine whether EB1 inhibits *Fo*c TR4 *in planta*, banana tissue culture plantlets were infected with *Foc* TR4 but only after a prior bacterization with an EB1. Using SEM, EB1 colonies were observed in grooves between root epidermal cells, indicating that the mechanism of entry of EB1 into roots occurs most probably *via* cracks, which also represent the major routes for phytopathogen to enter into plants (Compant et al., 2010; de Zélicourt et al., 2018). Correspondingly, the deformed hyphae of *Foc* TR4 and alleviated host damage were observed *in planta* due to the inoculation of EB1, suggesting that penetration of *Foc* TR4 through cellophane membranes and invasion of banana tissue were impaired upon co-inoculation with EB1. Therefore, EB1 might occupy the ecological niches and nutrition rapidly and act as an extracellular barrier for the host plant for blocking the pathogen invasion (Gao et al., 2010; Shafi et al., 2017; Dubey et al., 2020). It is noteworthy that the response of banana plants toward EB1 bacterization in the rooting stage was maintained and further amplified in the pot experiment. Plants whose roots had been pre-inoculated with EB1 at the rooting stage showed significantly higher survival rates and better growth states compared with those inoculated only with *Foc* TR4. Using antiSMASH, 12 BGCs responsible for the synthesis of 8 secondary metabolites including surfactin, bacilysin, bacillibactin, difficidin, fengycin, bacillaene, macrolactin, and butirosin have been identified in the genome of EB1. Surfactin and fengycin have been widely characterized to mediate biofilm formation and root colonization processes, which are suggested to have a role in plant development and growth promotion (Aleti et al., 2016; Berlanga-Clavero et al., 2022). In addition, putative genes involved in the production of indole-3-acetic acid (IAA), spermidine, and polyamine, which are related to plant growth-prompting activity, have also been discovered in the genome of EB1 (Xie et al., 2014; Zaid et al., 2022). Thus, our results have demonstrated the promising application of endophytic antifungal strains in agriculture to breed “microbe-optimized crops”.

Different from the fighting to the death in pathogen and host relationship, recent conceptual and experimental framework has indicated that beneficial endophytes usually can evade plant defense and reach a stable harmonious commensalism with the plant (Sessitsch et al., 2012; Deng et al., 2019; Yu et al., 2019). To figure out the role EB1 plays in the three-way interactions with the host plant immunity and the fungal pathogen, expressions of genes known to be markers of plant defense signaling pathways including SA-mediated *NPR1* and *PR1* as well as JA-mediated *LOX* and *MYC2* were analyzed (Mhamdi, 2019). It is found that the expressions of these genes were stronger in plants with EB1 pretreatment and *Foc* TR4 infection than that in plants with pathogen infection only which is in accord with previous research (Chandrasekaran and Chun, 2016; Nie et al., 2017). Similarly, inoculation of wheat with endophytic bacterium *Stenotrophomonas rhizophila* SR80 increased the expressions of a range of genes in SA and JA signaling pathways, but only when the *F. pseudograminearum*, the causal agent of Crown rot disease, was present (Liu H. et al., 2020). Our findings suggested that EB1 plays a key role in the interactions with the host plant immunity and the fungal pathogen *via* a mechanism that enhances plant defense and growth (Khare et al., 2018). A few studies mentioned that beneficial microbes can quench plant immune responses by downregulating the expression of the microbial-associated molecular patterns (MAMPs) (Bardoel et al., 2011; Zamioudis and Pieterse, 2012), producing the MAMPs with a low-elicit ability (Trda et al., 2014), or minimizing the stimulation of plant defensive response (Liu et al., 2018; Deng et al., 2019). Accordingly, the whole-genome annotation data suggest that EB1 contains multiple genes that encode key components that function by these mechanisms. Combined with the localization and *in vitro* data, these observations suggest that EB1 forms a symbiotic relationship with banana plants and efficiently wards off the invasive of *Foc* TR4 *in planta* inferring the adaptability and potential of the banana tissue culture plantlets bio-primed with EB1 could be a promising biological solution for the management of Fusarium wilt of banana.

## Conclusion

Our current study focused on providing a comprehensive understanding of the endophytic strain *Bacillus velezensis* EB1 isolated from a healthy banana plant in a wilt-diseased banana field and exploring its potential application in tissue culture plant of banana for the environmental sustainability management of Fusarium wilt based on its strong antagonistic effects against the devastating fungal pathogen *Foc* and mutualistic functional roles with banana plants. To realize large-scale implementation of microbial strains in agricultural practice, new strategies for successful delivery of BCAs into plant under field conditions are needed. Therefore, in the future, we intend to (1) understand the underlying molecular mechanisms of the beneficial effect of EB1 on the growth and stress tolerance of banana plants, (2) isolate more efficient, multifunctional, stress tolerant microbes and design an artificial disease suppressive synthetic community (SynCom) which comprised by multiple microbial strains rather than mono-strain inoculums to take advantage of functional complementarity to mimic a natural disease-suppressive community in plants, and (3) develop bioformulations for sustainable application of endophytic microbes in plant tissue culture. New strategies for the successful delivery of BCAs into the plant under field conditions are needed to realize the large-scale implementation of microbial strains in agricultural practice. The introduction of endophytic microbes, as a probiotic material that enhances plant growth as well as induces defense responses of plants to cope with stress, into tissue-cultured banana plantlets, could be a novel and stable biological control method to protect bananas from *Foc* infection.

## Data availability statement

The datasets presented in this study can be found in online repositories. The names of the repository/repositories and accession number(s) can be found in the article/Supplementary material.

## Author contributions

DX and SL: conceptualization. DX, XY, BL, and YC: experimentation. DX, XY, and CL: review and drafting. SL and CL: validation and statistical analysis. All authors contributed to the article and approved the submitted version.
