# Supplementary material for: Bio-priming of banana tissue culture plantlets with endophytic Bacillus velezensis EB1 to improve Fusarium wilt resistance
Source: Front Microbiol. 2023 Mar 16;14:1146331. doi: 10.3389/fmicb.2023.1146331 (PMC10064985; doi:10.3389/fmicb.2023.1146331)
Supplement: Supplementary file 1 [file Data_Sheet_1.DOCX]

Supplementary Material

Bio-priming of banana tissue culture plantlets with endophytic *Bacillus velezensis* EB1 to improve Fusarium wilt resistance

**Dandan Xiang^1^, Xiaofang Yang^1,2^, Bojing Liu^1^, Yuanqi Chu ^1,2^, Siwen Liu^1^, Chunyu Li^1*^**

*** Correspondence:** Chunyu Li: lichunyu@gdaas.cn

# Supplementary Figures and Tables

## Supplementary Figures


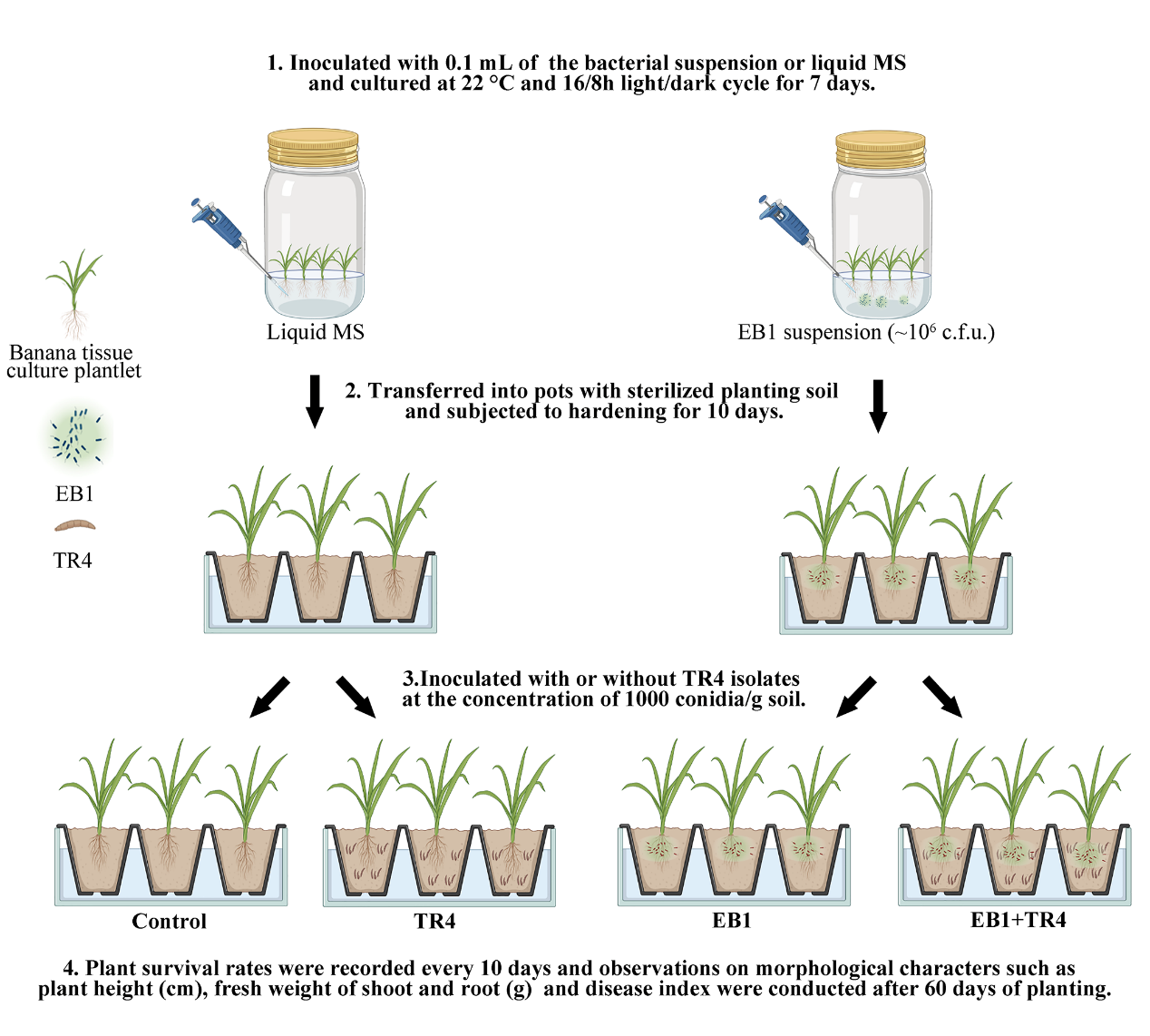


**Supplementary Figure 1.** Schematic representation of EB1biopriming and TR4 inoculation method used in the pot experiments.


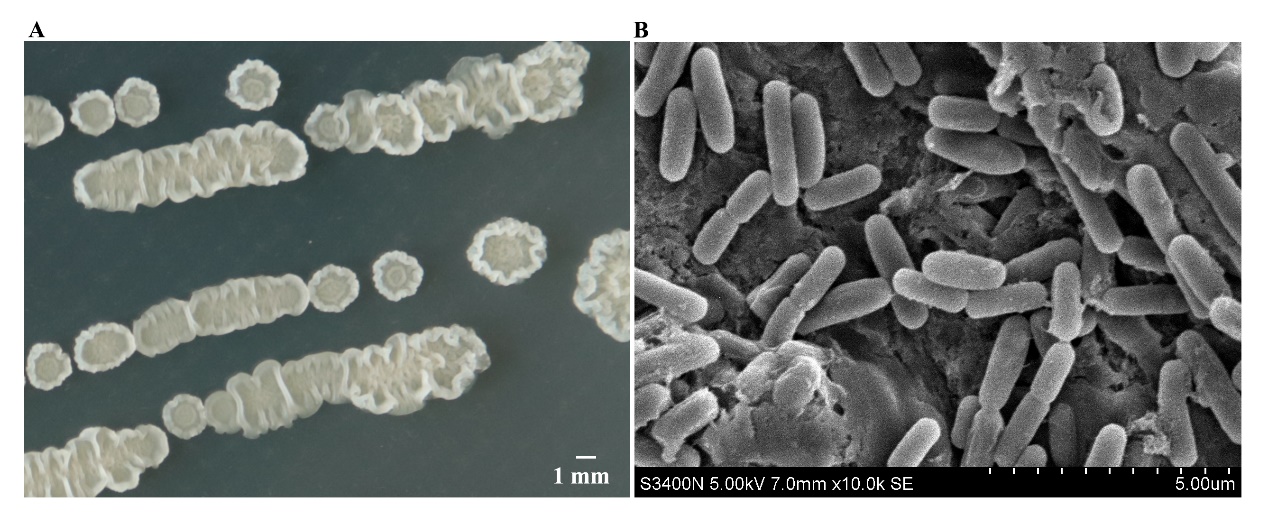


**Supplementary Figure 2.** Morphology of endophytic bacterium strain EB1. (A) Colony morphology of EB1 grown for 24 h on LB agar plate. (B) Bacterial cell morphology under a scanning electron microscopy (SEM).


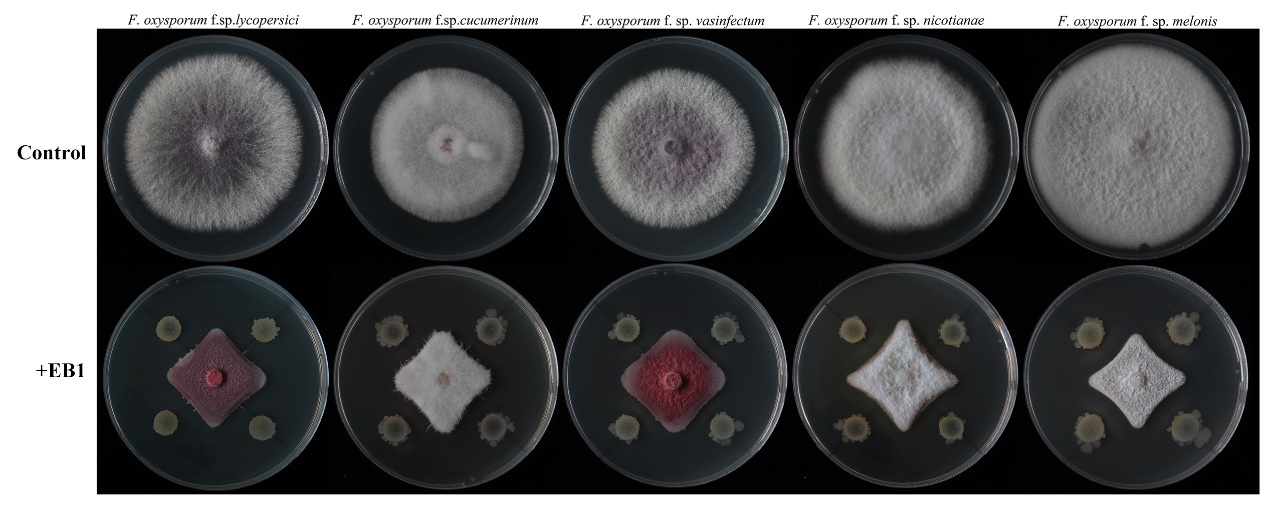


**Supplementary Figure 3.** EB1 is a broad-spectrum endophytic strain shows strong inhibitory activities against Fusarium pathogens.


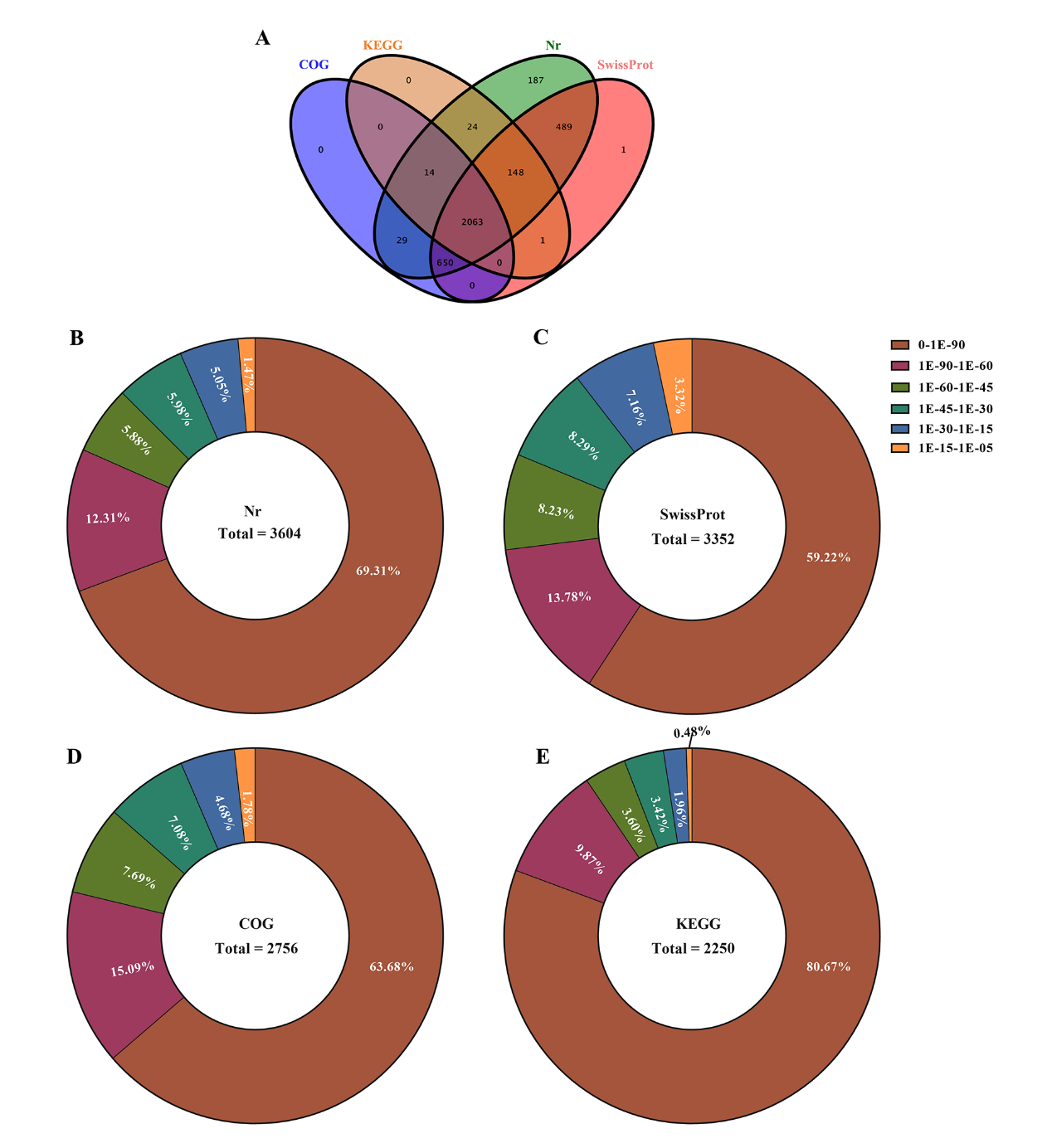


**Supplementary Figure 4.** Function annotation were performed by BlastX against the protein databases NR, SwissProt, COG and KEGG (E-value < 1.0E-5). (A) The Venn diagram between NR, Swissprot, COG and KEGG; the annotated gene numbers were exhibit in the related areas. (B-E) The E-value distribution of Nr (B), SwissProt (C), COG (D) and KEGG (E) annotation results.*


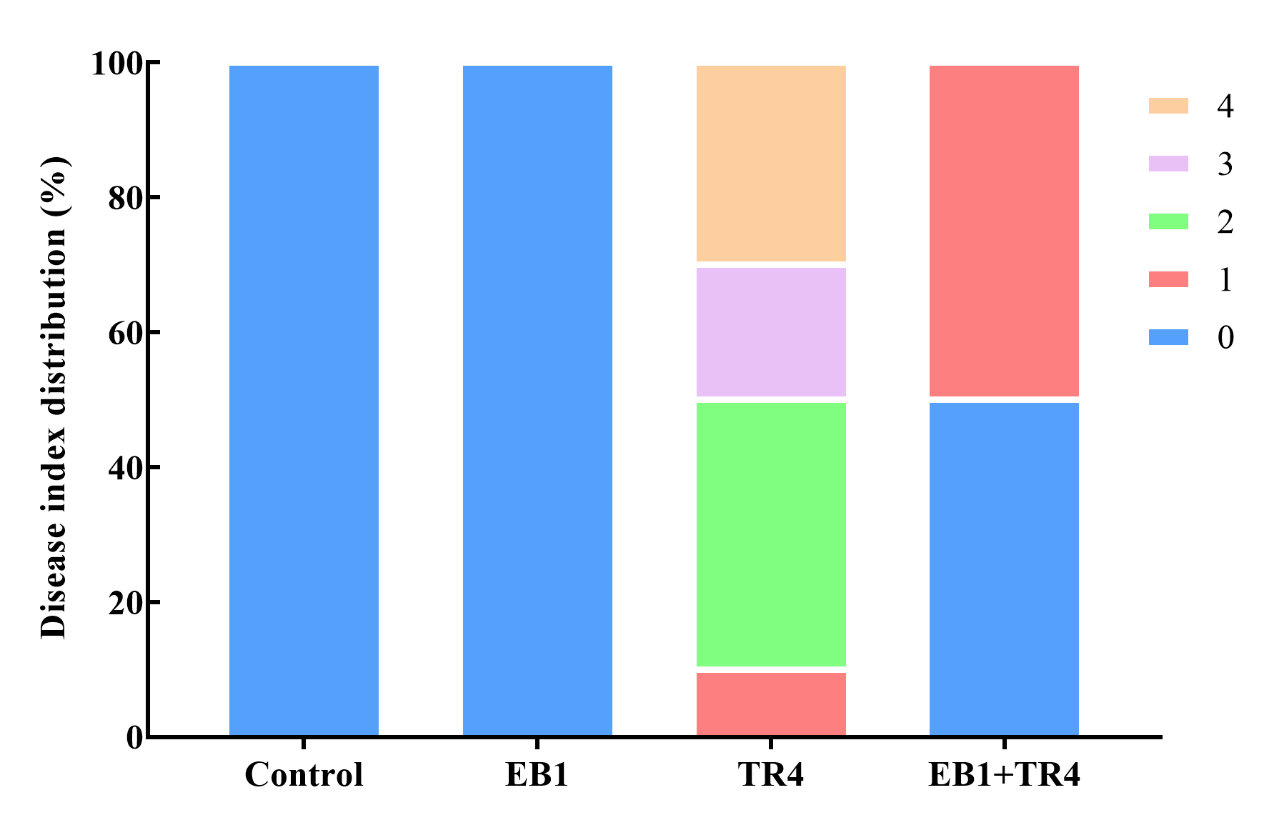


**Supplementary Figure 5.** Disease index of acclimatized banana plants in greenhouse experiment. Disease severity was assessed on a four-scale rating standard.

## Supplementary Table

**Table S1. Primer sequence used in the present study.**

| **Gene** | **Primers** |  |
| --- | --- | --- |
| *NPR1* | F: 5' GGAGATCCACAAGTAGGTGAAGC 3' | R: 5' AGTCTTGCCAGAGCAACTCG 3' |
| *PR1* | F: 5' TCCGGCCTTATTTCACATTC 3' | R: 5' GCCATCTTCATCATCTGCAA 3' |
| *LOX2* | F: 5' CTTCCTCACCACCCTCATCT 3' | R: 5' CCACGACCGCCATGAACTTG 3' |
| *MYC2* | F: 5' CGGATCTACCGACGTGGTCT 3' | R: 5'AGCGTCCGGAGAGCTAAAGT 3' |
| *qTUB* | F: 5' AGCAACTCCTACTTTTGGCGATC3' | R: 5' TGAGGGCCCTGTATTGCTGG3' |
